# A novel collaborative e-learning platform for medical students - ALERT STUDENT

**DOI:** 10.1186/1472-6920-14-143

**Published:** 2014-07-14

**Authors:** Tiago Taveira-Gomes, Areo Saffarzadeh, Milton Severo, M Jorge Guimarães, Maria Amélia Ferreira

**Affiliations:** 1 Department of Medical Education and Simulation, Faculty of Medicine of the University of Porto, Porto, Portugal; 2 ALERT Life Sciences Computing, Vila Nova de Gaia, Portugal; 3 University of California Irvine School of Medicine, Irvine, USA

**Keywords:** Medical education, Computer supported collaborative learning, E-learning, Information management, Memory retention, Computer-assisted instruction, Tailored learning, Student-centered learning, Spaced repetition

## Abstract

**Background:**

The increasing complexity of medical curricula would benefit from adaptive computer supported collaborative learning systems that support study management using instructional design and learning object principles. However, to our knowledge, there are scarce reports regarding applications developed to meet this goal and encompass the complete medical curriculum. The aim of ths study was to develop and assess the usability of an adaptive computer supported collaborative learning system for medical students to manage study sessions.

**Results:**

A study platform named ALERT STUDENT was built as a free web application. Content chunks are represented as *Flashcards* that hold knowledge and open ended questions. These can be created in a collaborative fashion. Multiple *Flashcards* can be combined into custom stacks called *Notebooks* that can be accessed in study *Groups* that belong to the user institution. The system provides a *Study Mode* that features text markers, text notes, timers and color-coded content prioritization based on self-assessment of open ended questions presented in a *Quiz Mode*. *Time spent studying* and *Perception of knowledge* are displayed for each student and peers using charts. Computer supported collaborative learning is achieved by allowing for simultaneous creation of *Notebooks* and self-assessment questions by many users in a pre-defined *Group*. Past personal performance data is retrieved when studying new *Notebooks* containing previously studied *Flashcards*. Self-report surveys showed that students highly agreed that the system was useful and were willing to use it as a reference tool.

**Conclusions:**

The platform employs various instructional design and learning object principles in a computer supported collaborative learning platform for medical students that allows for study management. The application broadens student insight over learning results and supports informed decisions based on past learning performance. It serves as a potential educational model for the medical education setting that has gathered strong positive feedback from students at our school.

This platform provides a case study on how effective blending of instructional design and learning object principles can be brought together to manage study, and takes an important step towards bringing information management tools to support study decisions and improving learning outcomes.

## Background

Medical education is an area of increasing complexity, considering the education goals of health professionals for the XXI century [1,2]. Successful medical learning requires a considerable time investment not only in the development of core and specific competencies, but also in the ability to transfer basic cognitive competencies to the clinical setting through the integration of personal experience and vast information sources [1,3].

Information management regards the ability to search, identify and integrate relevant information that can be further used for critical reasoning in clinical practice [4] and is currently one of the most compelling challenges facing medical students.

### Approaches to enhance learning

In many settings, information is not effectively managed during learning. The demanding learning process frequently drives students to retain knowledge to meet course goals instead of strengthening competence development [5]. According to the Adaptive Character of Thought (ACT-R) theory “time on task” is the most important factor for developing lifetime competence [6]. As the amount of knowledge to learn increases, how well time is managed in the learning processes becomes key [6]. Cognitive load theory postulates three types of cognitive load: (a) intrinsic load is the net result of task complexity and the learner expertise; (b) extraneous load is caused by superfluous processes that do not directly contribute to learning; (c) germane load is accounted by learning processes handling intrinsic cognitive load [7]. Studies have been carried to identify design guidelines and benefits of this theory in health sciences education [6,8-13].

Spaced-repetition, a learning approach that focuses on reviewing content multiple times over optimized time intervals is one of the most effective ways to improve long-term retention [14-18]. While evidence-based principles for instructional design are abundant, they are infrequently incorporated into the educational setting in a consistent and deliberate manner [19].

### Learning objects

The way in which content can be organized in order to optimize learning has also been extensively studied [4,13,20-23]. Learning objects, groupings of instructional materials structured to meet specific educational objectives [23], define a set of guidelines to make content portable, interactive and reusable, [23-27] therefore enhancing and tailoring learning [26]. They may facilitate adaptive learning by offering the chunks of content that the learner needs in order to achieve an accepted level of competence.

Other authors have identified the need to simplify the learning object authoring process to gain wider acceptance and use [28]. Additionally, the design of appropriate and effective technologies must take into account individual differences in learning, through systems that adapt based on individual progress and performance or through explicit choices made by the learner [29].

Students need tools to help retain knowledge for longer periods and easily identify materials with lesser retention rates [18]. This goal may be achieved by providing learners with personal insight on their learning effectiveness, using personal and peer progress data based on self-assessment results [26].

### Computer supported collaborative learning

Currently, web applications can be a valuable tool to reach information management goals. The application of new learning technologies that has emerged as a main stream in medical education [30] is known to simplify document management, communication, student evaluation and grading [31]. However, these tools focus mainly on maximizing efficiency of administrative teaching and have little in consideration the learning tasks directed at students.

Additionally, over recent years there has been a shift in medical education where traditional instructor-centered teaching is yielding to a learner-centered model [28,32]. With the advent of social media tools that allow for collaboration and community building it is becoming more common for students to create and share materials on-line [25,33]. However, these materials are often not validated or reviewed by teachers [34,35] and may decrease learning effectiveness as the student will need to browse, filter and validate relevant information from numerous and often conflicting information sources [36].

CSCL can add an instructor role to the learner-centered model. It can place learners in control of their own learning and transforms the role of a teacher from the sole-provider of information to a facilitator of knowledge acquisition [28,35] promoting greater learning satisfaction [17,37]. This type of approach usually takes place in asynchronous collaboration settings where students and teachers can collaborate at different times [37-39]. Despite this potential, little evidence of effectiveness on using such tools in the health professions has been gathered [17,40].

Effective information management during the learning process may be achieved through adoption of computer supported collaborative learning (CSCL) systems that provide validated content in the form of learning objects, allow student self-assessment and display tailored feedback that can be used to support study management. This data should direct further exploratory or limited learning approaches, so that knowledge acquisition may be benefited at the same time information management competences are developed.

The present study aims to develop and assess the usability of an adaptive CSCL system that helps making decisions regarding personal learning process. So far, existing studies regarding such systems were built to be applied in specific medical knowledge fields [8,41-44]. To our knowledge no system has been built to be of application to medical curricula in general [45].

## Implementation

### Technologies

**Figure 1:**
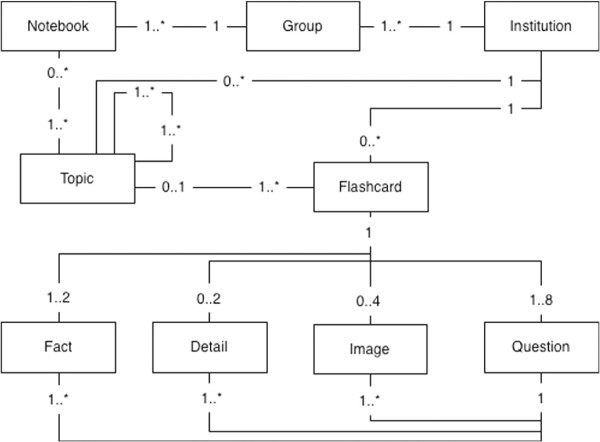
**Simplified Entity relationship UML diagram.** A simple UML diagram that specifies relationships between the main application objects. Multiple *Notebooks* belong to a *Group*, and multiple *Groups* belong to an institution. An institution has multiple topics and *Flashcards*. A *Notebook* may hold multiple topics that are associated to multiple *Flashcards*. Multiple topics can also belong to a broader topic. A *Flashcard* can be composed of one or two facts, up to two description items, up to four images and one to eight questions. Multiple questions can be associated to a *Fact*, *Description* or *Image*.

The present application was built in accordance to current web standards. The user interface was built using HyperText Markup Language (HTML), Standard Vector Graphics (SVG) and JavaScript. The application layer of the system was built using JAVA technology over the Play!Framework version 1.2. The database layer was built using ORACLE systems. The data model is described using a simplified UML diagram in Figure 1. A simpler version of the application was developed for the iPhone but will not be discussed in this paper.

### Content structure

Content was required to be stored reusable blocks that would allow building of higher order learning blocks as well as assessing knowledge. Knowledge assessment was carried out using open ended questions. The smallest learning block was named *Flashcard*, and was composed of information on one side and open ended questions on the other. Each *Flashcard* contained up to 8 knowledge pieces named *Fact*, *Description* and *Image*. Questions can be associated to each of these pieces individually. Each piece would therefore serve as the answer to one or more questions. Since content re-usability was paramount, a *Flashcards* categorization system was implemented using Medical Subject Headings (MeSH) from the United States National Library of Medicine.

Aggregation of *Flashcards* in higher order structures was required to achieve meaningful learning goals. That would require creating custom aggregations of *Flashcards* of different MeSH topics. Topic and *Flashcard* order should be arranged according to the learning goal. We named these custom aggregations *Notebooks*.

In order for students and teachers to create and share content, *Groups* were created. *Groups* reside within institutions. Therefore, users from a given institution could access its *Groups*. A universal institution was created in order to allow all users to create and share content globally.

### Learning tools

User information regarding study metrics needed to be collected for study management. *Time spent studying* and *Perception of knowledge* were the two identified metrics required to meet this goal (Table 1). *Perception of knowledge* refers to student self perception of how well knowledge could be recalled when an open-ended question is presented. This data allowed computation of *Flashcard* study priority levels. These features were collected and presented in different sections: one devoted to study - *Study Mode*; another devoted to self-assessment - *Quiz Mode*; and a section devoted to analysis of performance metrics per *Notebook* - *Notebook Dashboard*.

**Table 1:** Variables measured by the system

**Name**	**Meaning**	**Measurement and presentation**
Study session count	The number of times a	The *Study Mode* provides a button that when clicked increments the study
	*Notebook* has been studied	session count for the *Notebook*.
Time spent studying	*Time spent studying* a	Each *Flashcard* provides a button to mark itself as studied. Each time that
	*Flashcard* for a study session	button is pressed, the time lapse since a previous click in any other
		*Flashcard* is added to the clicked *Flashcard* time for the current study session.
		*Time spent studying* is presented as the cumulative time for all sessions per
		*Flashcard* in a chart. It is represented as the proportion of the *Flashcard* time
		to the global *Notebook* time on the sunburst chart.
Perception of knowledge	The student self perception of knowledge	The student is presented an open ended question that requires recalling the
	regarding a *Flashcard* question.	knowledge to answer it. After recalling the question the student can see the
		answer and assess the quality of his recall using a 4-point likert scale.
		*Perception of knowledge* is presented as the average for a given *Notebook* or
		per Topic. It is represented as a percentage of the best possible
		*Perception of knowledge* for a *Notebook*.

### System usability and adoption surveys

System usability and feature usefulness of the *Study Mode*, *Quiz Mode* and *Notebook Dashboard* was assessed using a group of 48 students from the Faculty of Medicine of the University of Porto (FMUP) and two on-line self-report questionnaires. Students from the 4th and 5th years of the medical course were randomly selected and contacted by email to participate in the study.

The study consisted of 2 classroom sessions (S1, S2) in consecutive weeks, with duration of 1 hour. Each student was provided a computer. The students were instructed to use the *Study Mode*, *Quiz Mode* and *Notebook Dashboard* to study and assess their knowledge on a *Notebook* about the Golgi Complex. The *Notebook* was created using pedagogical materials provided by the Department of Cellular and Molecular Biology of FMUP.

During S1 students had 10 minutes to register in the platform. A 2 minute explanation of how the *Study Mode*, *Quiz Mode* and *Notebook Dashboard* worked was given to students before they used the application. All doubts were clarified. The students then spent 20 minutes on *Study Mode*, 15 minutes on *Quiz Mode* and 5 minutes on the *Notebook Dashboard*. After that time the students completed an on-line survey regarding system usability and tool usefulness. Students left the room only after all students completed all tasks.

During S2 students spent equal amounts of time on the *Study Mode*, *Quiz Mode* and *Notebook Dashboard*. At the end of the session, the system usability and tool usefulness survey was filled again and an additional survey regarding willingness to adopt the system as a reference tool was also completed.

The 3 surveys consisted of a set of objective statements regarding personal experience. Student agreement to each of the items was assessed using a 4-point likert scale: 1 - full disagreement; 2 - partial disagreement; 3 - partial agreement; 4 - full agreement.

Paired sample t-test was used to compare differences in the system usability and tool usefulness survey answers between the two sessions. Significance level was fixed at 0.05.

This study was approved by the Faculty of Medicine University of Porto/*São João* Hospital Ethics Committee in compliance with the Helsinki Declaration.

## Results and discussion

The platform was implemented as a free web application named ALERT STUDENT. Table 2 provides an outline of how learning objects principles were implemented in the system and Table 3 provides detail on how several instructional design features were implemented.

**Table 2:** Implementation of learning object principles

**Principle**	**Description**	**Implementation**
Stand alone	Learners can use a single learning object to	Each *Flashcard* encloses a small learning outcome. Combination of
	achieve a specified learning outcome.	*Flashcards* into *Notebooks* allow achievement of broader learning outcome.
Reusability	Learning objects can be used by diverse groups	*Flashcards* created for a given *Notebook* can be reused to create other
	of learners in a variety of educational situations.	*Notebooks* for different learning situations (eg.: within different *Groups*).
Interactivity	Each learning object requires an interactive	*Flashcards* and *Notebooks* require learners to highlight, take notes and self
	response from the learner.	assess their knowledge using features of the *Study Mode* and *Quiz Mode*.
Aggregation	Learning objects can be linked into larger collections	*Flashcards* can be liked into larger collections called *Notebooks*. *Notebooks*
	to form lessons, modules, or courses.	can be linked into larger collections by using *Groups*.
Interoperability	A learning object can be used with appropriate	*Flashcards* and *Notebooks* can be accessed on-line in any computer or using
	“plug-ins” by multiple software applications and on	the mobile application for the iPhone. The application interface that allows
	a variety of computers and e-learning platforms.	communication with the iPhone also allows integration with external
		applications.
Accessibility	A learning object must be tagged with standardized	*Flashcard* are cataloged using MeSH terms and can be searched within the
	indexing information (metadata) that allows it to be	application by using these terms.
	easily found by course designers, educators,	
	learners, and evaluators.	

Descriptions are adapted from Ruiz et al. [23].

**Table 3:** Implementation of instructional design principles

**Principle**	**Implementation**
Coherence principle: eliminate extraneous material	Splitting of content into facts and description components. Ability to hide tools in *Study Mode*.
	Ability to resume from where last study session was left.
Signaling principle: highlight essential material	Bold typeface for facts. Text marker feature. *Flashcard* color-coded study prioritization based
	on learner *Perception of knowledge*.
Pre-training principle: provide pre-training in names	
characteristics of key concepts	*Notebooks* with key *Flashcards* can be provided before more advanced *Notebooks* are studied.
	Introductory *Flashcards* can be added to more advanced *Notebooks*.
Segmenting principle: break lessons into	
learner-controlled segments	*Flashcard* break *Notebook* content into learner controlled segments
Multimedia principle: present words and pictures	
rather than words alone	*Flashcards* support both text and images

Principles enumerated from Mayer et al. [46].

### *Groups*

**Figure 2:**
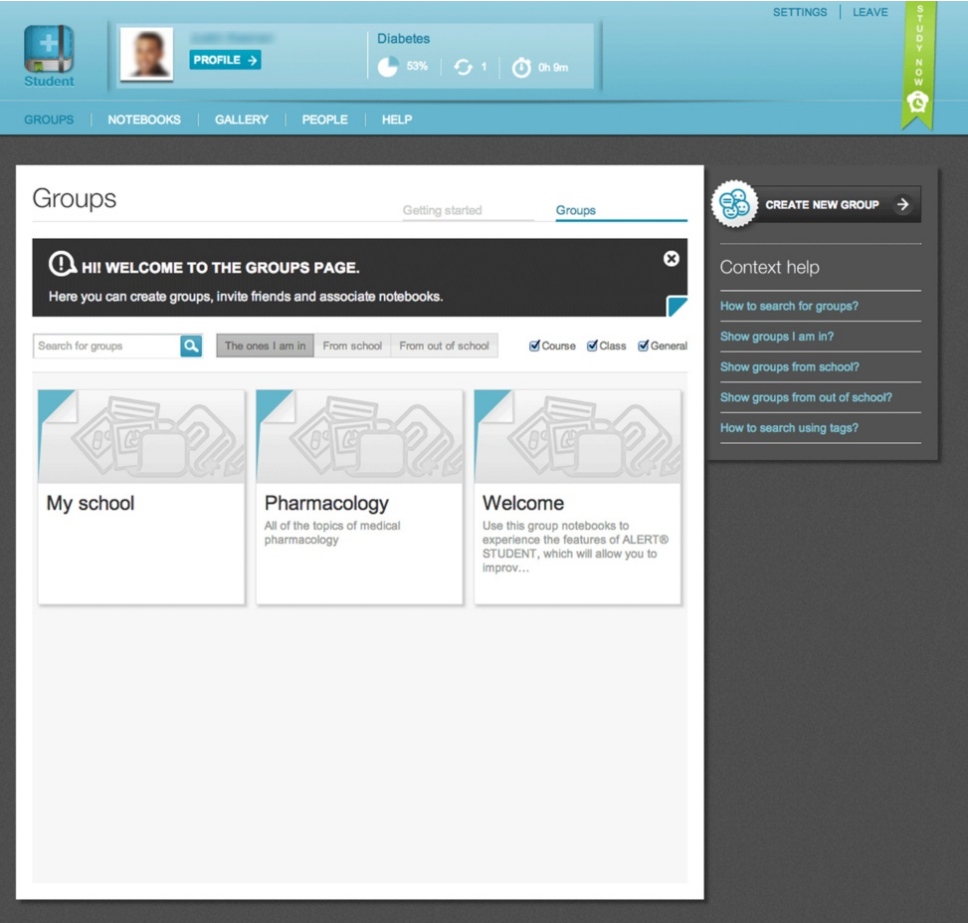
**User *Groups* screen.** A list of *Groups* for a given user is displayed.

The application has a section devoted to *Groups* (Figure 2). This section consists of a page listing all *Groups* and specific *Group* pages. The list page allows browsing *Groups* using search by name, tags and filtering by belonging institution. The *Group* page was divided into 4 sections: (a) *Group* wall for posting and commenting; (b) member’s page where *Group* administrators can manage members; (c) *Notebook* page that holds *Notebooks* and allows creation or editing; (d) *Group* profile section where non-members can see the *Group* summary.

*Groups* allow a closed environment approach where students can interact with a defined set of users and content for a given learning goal. This is similar to the wiki or blog scenario where administrators limit registration and editing privileges to selected users [25]. Allowing *Flashcards* within a *Group* to be available to other *Groups* of the same institution facilitates content sharing within the institution. This helps to reduce content redundancy, allows faster content creation and allows new *Notebooks* to be created using previously studied *Flashcards*. This may lessen intrinsic cognitive load by reducing the exploratory component involved in learning new redundant materials, hence increasing learning performance [31].

### *Notebooks*

**Figure 3:**
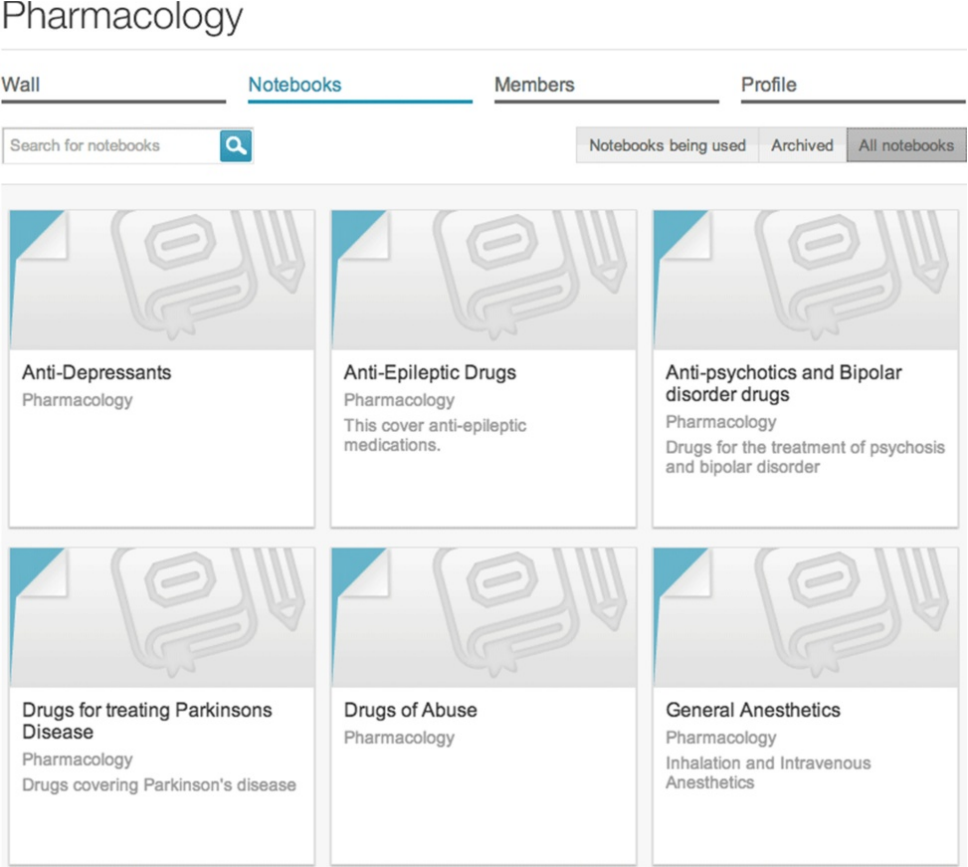
**User *Notebooks*.** A list of the *Notebooks* for a given user is displayed.

**Figure 4:**
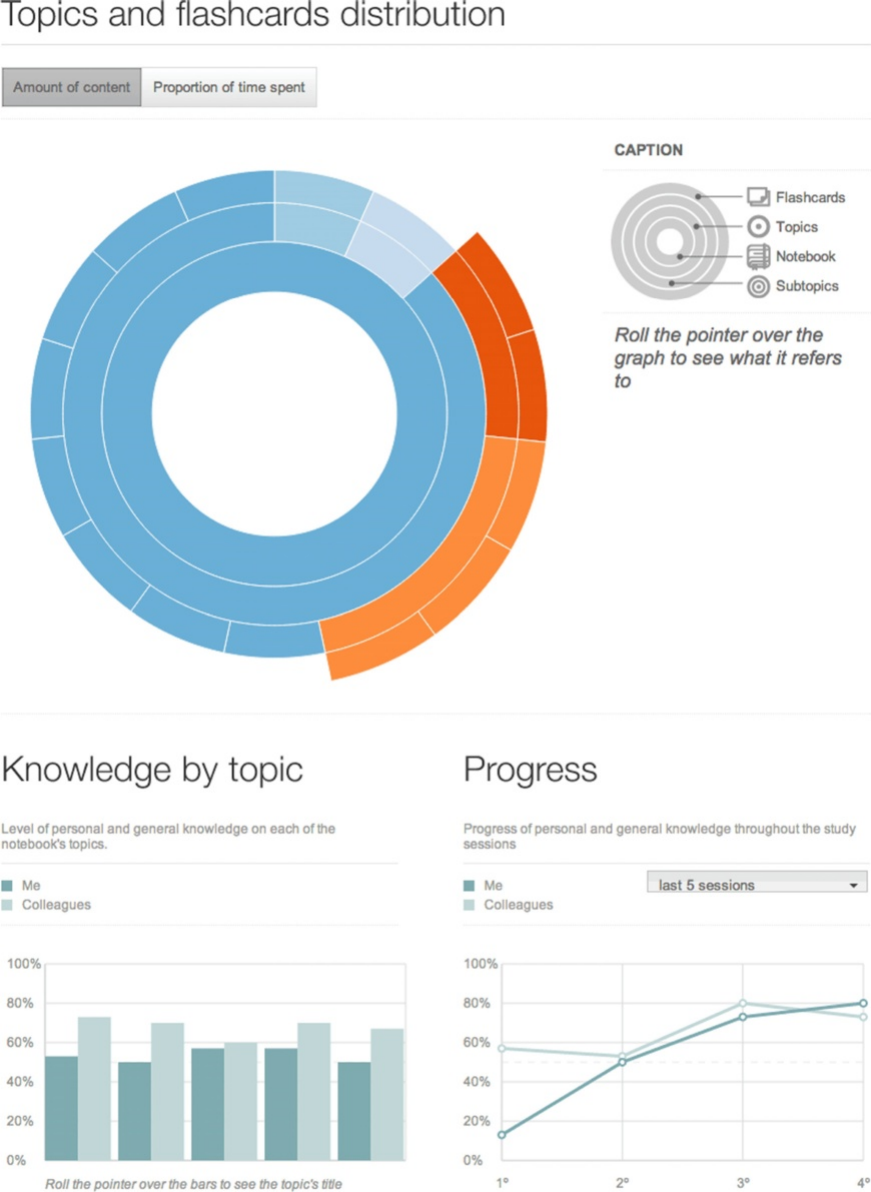
***Notebook *Dashboard.** The sunburst chart represents the topic and *Flashcard* distribution. The toggle button switches the configuration between *Flashcard* size (given by the number of characters) and *Time spent studying* on a *Notebook*. The bar chart on the left depicts *Perception of knowledge* per topic, for the user and its peers. The line chart on the right is represents *Perception of knowledge* per quiz session for the user and its peers.

**Figure 5:**
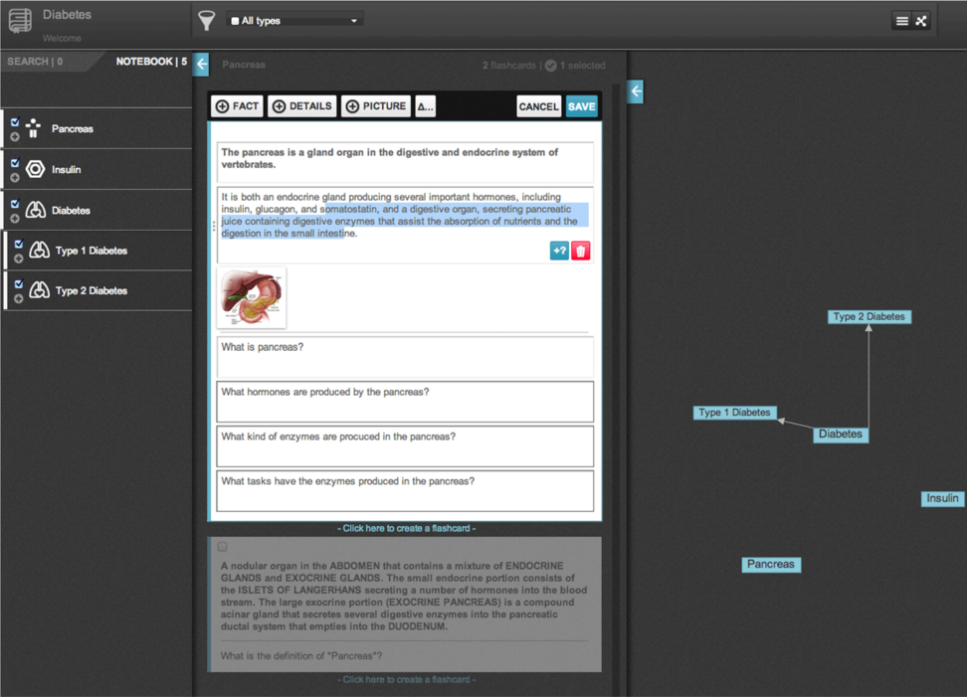
***Notebook *editor.** Topics can be browsed on the left column on the search tab. Checked topics become part of the *Notebook* and become available on the notebook tab. The center column displays *Flashcards* for the selected topic. Checked *Flashcards* become part of the *Notebook*. New *Flashcards* can be created on any topic. On the right MeSH relationships between topics are represented using a graph that can be used to navigate topics.

*Notebooks* can be accessed through *Group* pages or through a global *Notebook* page. Both pages provide search and filter features. (Figure 3) The *Notebook Dashboard* shows overall information and study statistics regarding personal study performance. Users can analyze *Flashcard* size and *Time spent studying* using a sunburst chart (Figure 4). A toggle button resizes each *Flashcard* representation to match either its character count or time taken. A bar chart plots *Perception of knowledge* per topic in two series. One series plots user *Perception of knowledge* while another plots mean peer *Perception of knowledge*. A line chart plots *Perception of knowledge* per quiz session in two series as well. One series plots user *Perception of knowledge* while another plots mean peer *Perception of knowledge* (Figure 4). The *Notebook* editor allows simultaneous creation of *Notebooks* by searching and selection topics and *Flashcards* available to be part of a *Notebook*. New topics and *Flashcards* can be created as well. A graph of MeSH topic relationships is also displayed and can be used to browse topics (Figure 5).

*Flashcards* allow content to be created in ways that match specific learning goals and can be reused with little effort to match other learning requirements. Though they are in accordance to the learning objects principles of stand-alone, reusability, interactivity and aggregation [23] (Table 2), the amount of context to build these type of learning objects must be balanced in a way that allows isolated usage in different settings as well as chaining with additional *Flashcards* in meaningful ways [26]. Enclosing little context in each *Flashcard* may lead to less articulated *Notebooks*.

*Flashcards* are supported by the cognitive load theory. Small chunks of self-enclosed knowledge decrease intrinsic cognitive load. Additionally, since *Notebooks* are combinations of *Flashcards*, they can orient learning in a simple-to-complex strategy that further decreases intrinsic cognitive load [6,9,47]. Furthermore, this process can be extended by refactoring multiple *Notebooks* into smaller summary *Notebooks* containing the most relevant *Flashcards* that leverage the same cognitive load principles further [47]. Performance data for overlapping *Flashcards* can be used to optimize study sessions in a new *Notebook* setting, which also applies to the principles of learning object re-usability, interactivity and aggregation [47] (Table 2).

The charts allow the student to take action on their study sessions based on *Time spent studying* and personal and peer *Perception of knowledge*. Previous works have shown that feedback play a key role in determining learning success [26], hence, insight into performance metrics may help build motivation to learn further.

### *Study Mode*

**Figure 6:**
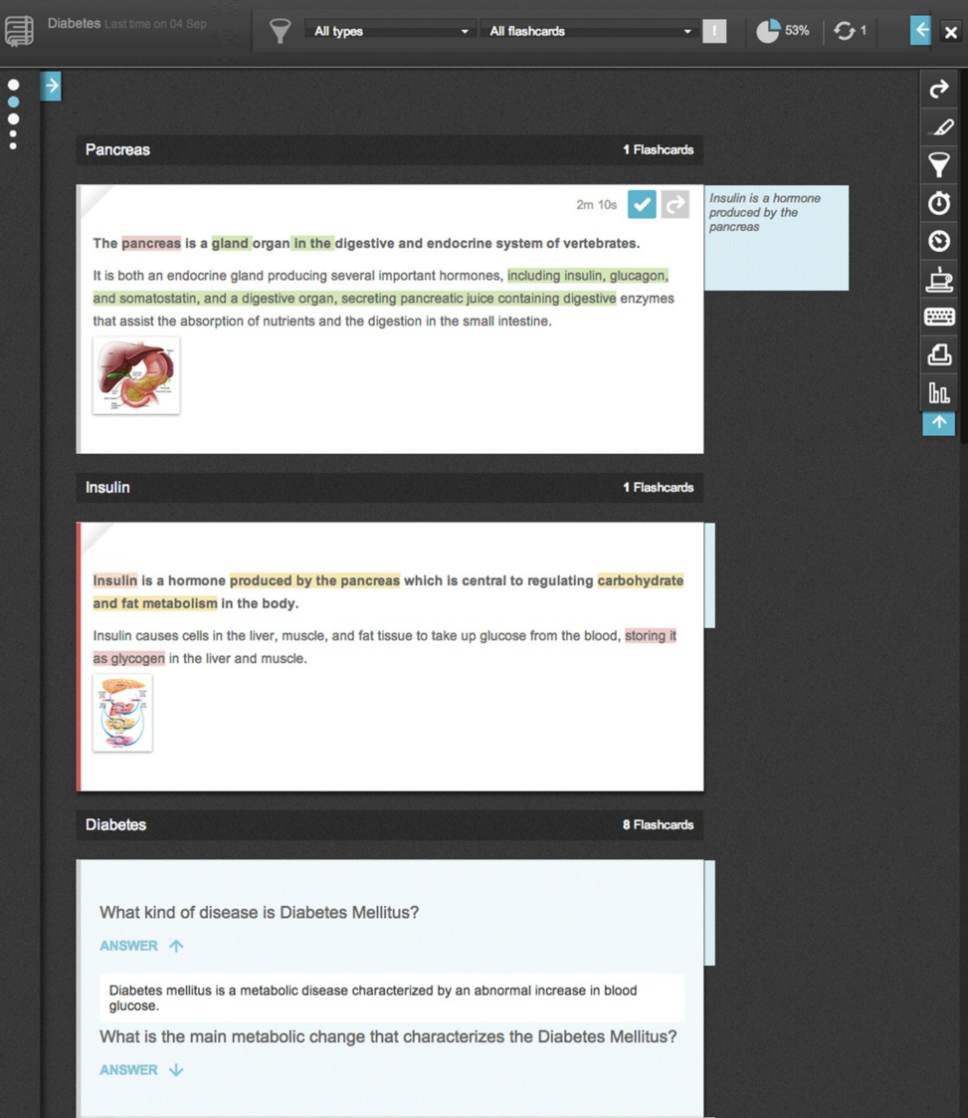
***Study Mode*.** The left column with circles represent the *Notebook* topic index. The blue circle represents the topic currently displayed. The top bar houses the content filters and progress status. Timers are also available but not shown. The bar in the right side is the actions bar, that houses *Flashcard* flipping, text marker, filter and timer toggle, pause mode, keyboard shortcuts list, print view and shortcut to statistics buttons. The third *Flashcard* displayed is flipped, showing questions and an answer.

The *Study Mode* allows *Notebook* study in an adequate digital environment, which minimizes sources of distraction (Figure 6). The dark colors used on the interface contrast with the white *Flashcards*, creating focus on the area of interest. The center displays the *Flashcards* stacked as a continuous piece of text. On the side, the index of topics is displayed. It also provides study progress metrics such as percentage of *Flashcards* studied, number of study sessions, time taken per session, total *Time spent studying* and *Time spent studying* on the previous session. *Flashcards* can be flipped one at a time or altogether to reveal the questions. *Flashcards* have a button to increment *Time spent studying* and can be removed from the *Quiz Mode* assessment by folding the top left corner with a simple click. Additionally, *Flashcards* have a colored bar on the side that expresses *Perception of knowledge*. All tool menus are collapsible to prevent distractions. Available tools include filters for *Flashcard* priority and category, a timer, a stopwatch, notes and text highlighters. Other tools present the keyboard shortcut guide and allow exporting the *Notebook* in.pdf format.

In order to increase reading speed, comprehension, and reduce fatigue from screen reading, spaced lines with a mean of 70 characters in length and large window height were used as mentioned in previous studies [48,49]. The ability to hide tools and the keyboard shortcuts further improves focus. *Flashcard* category and priority filters allow learning sessions to be tailored to personal goals effectively. These features may help reduce extraneous cognitive load related to content navigation tasks and interface visual noise [11,47]. Flipping the *Flashcard* column provides a tailored “content-and-question” oriented study environment. The ability to resume study sessions from the point that they were last left, further reduces extraneous cognitive load by decreasing distance to the required point of focus [11,47].

### *Quiz Mode*

**Figure 7:**
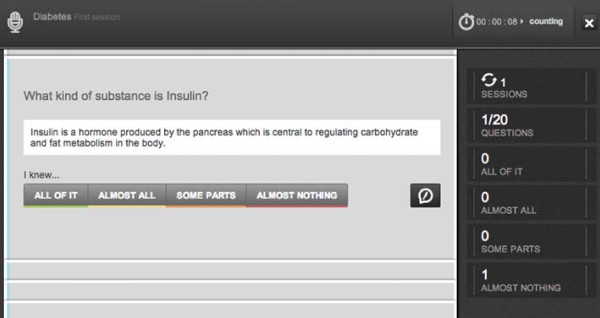
***Quiz Mode*.** A question card is represented along with the answer. *Perception of knowledge* is graded using the set of four buttons shown. The rightmost button reporting of errors to the *Notebook* owner. The column on the right tracks student progress.

The *Quiz Mode* is the section devoted to self-assessment (Figure 7). It takes the *Flashcards* of a *Notebook*, and selects a set of *Flashcard* questions that are presented one at a time. For each question the user should recall the required knowledge. Afterwards the user reveals the *Flashcard* section that answers the question and grades *Perception of knowledge*, the quality of the user recall, using a 4-point likert scale. After grading *Perception of knowledge*, the system shows another question. The student also has the option of reporting the *Flashcard* to the *Group* administrators when inaccuracies are found. After the evaluation step, another card is shown. The system displays student progress and the number of questions rated per grade. When the user finishes the Quiz, statistics about the *Time spent studying* on each session are presented. The student can also review the *Flashcards* for the questions with the lowest *Perception of knowledge*. Questions are chosen so that all flashcard elements are assessed. If more than one question is available for a given content piece, then the system will chose either the hardest question if there are previous ratings, or will pick a question at random. Global *Perception of knowledge* for each *Flashcard* is computed by calculating a weighted average of the last three sessions *FlashcardPerception of knowledge*. The session *Perception of knowledge* for a *Flashcard* is calculated by averaging the results for every question answered for the *Flashcard* in that session.

The *Quiz Mode* is essential for the system to compute *Perception of knowledge*. Because each *Flashcard* may have multiple questions regarding the same content piece, the *Quiz Mode* is able to use the questions with lowest *Perception of knowledge*. This provides a means to assess knowledge using questions that are most difficult thereby tailoring memory retention needs. This is also in accordance to the intrinsic cognitive load strategy of low-to-high fidelity tasks because as the student progress, questions representing harder tasks will be preferentially selected [47]. Spaced repetition promotes development strengthening of long-term memory schemata acquired during previous contacts with the *Flashcards*. This will reduce the amount of elements that will be dealt with using working memory, thus reducing cognitive load and allowing additional focus on the recall process [47]. The way the user grades *Perception of knowledge* is, however, subject to affective factors. Users may feel inclined to overrate their *Perception of knowledge* thus decreasing the beneficial effect of the system [50]. Although self-assessment questions are demonstrated to positively affect learning outcomes [16,19,50-53], it remains unknown whether self-reported evaluations correlate with exam grades. This question system has as primary goal to allow self-assessment of simple recall questions. Integrated reasoning questions that require integration of multiple pieces of knowledge are a second and more important step, that the authors intend to develop in the future.

This system implements other features, such as a content repository for FMUP students, the ability to present the *Notebooks* using full screen *Flashcards* and, a picture gallery, however these are not presented as their purposes are distinct from the goals of this work.

### System usability and adoption surveys

The student participation rate was 100% as all of the 48 students randomized to take part in this work accepted to participate. All students completed the two sessions. The score for all items on the survey regarding system usability and tool usefulness (Tables 4 and 5) approached 3.5 (partial to full agreement) in both sessions and overall there were no significant differences between sessions. Both surveys have shown that students generally agreed that the tools provided were useful and simple and were willing to use them as a privileged element for their medical education.

**Table 4:** System usability and tool usefulness survey

**n**	**Item**	**S1**	**S2**	**p**
1	It was easy to study using the computer	3.21 (0.69)	3.38 (0.61)	0.04
2	The *Study Mode* was easy to use and understand	3.68 (0.52)	3.81 (0.40)	0.06
3	The division of content using topics and			
	*Flashcards* was easy to understand	3.64 (0.53)	3.68 (0.47)	0.60
4	The division of *Flashcards* into Facts, Details,			
	Images and Questions was easy to understand	3.60 (0.58)	3.77 (0.43)	0.04
5	The division of *Flashcards* into Facts, Details, Images			
	and Questions helped to understand the key			
	information to memorize	3.43 (0.58)	3.45 (0.72)	0.84
6	The information on the *Flashcards* was simple and clear	3.62 (0.49)	3.60 (0.54)	0.80
7	The *Flashcards* were presented in a logical sequence			
	that facilitates learning	3.34 (0.67)	3.43 (0.65)	0.29
8	It was easy to find the *Flashcards* I wish to study using			
	the *Flashcard* filters	3.38 (0.61)	3.38 (0.61)	1.00
9	The highlighter and the notes are useful features	3.66 (0.64)	3.72 (0.54)	0.41
10	The Questions on the *Flashcards* were easy to understand	3.34 (0.73)	3.45 (0.65)	0.37
11	The Questions were helpful to help me assess my knowledge about each subject	3.62 (0.61)	3.62 (0.53)	1.00
12	I could easily find the matching Answer to the Question in the *Flashcard* Component box	3.53 (0.58)	3.55 (0.48)	0.20
13	The order in which the Questions were presented did not affect my focus on answering	3.34 (0.90)	3.32 (0.69)	0.86
14	Without these tools I would not be able to obtain a similar acquired knowledge result	3.30 (0.81)	3.00 (0.83)	0.02

S1 and S2 refer to session 1 and session 2. The tasks performed were the same on both sessions. For columns S1 and S2 the values represent mean and standard deviation. Student agreement to each of the items was assessed using a 4-point likert scale: 1 - full disagreement; 2 - partial disagreement; 3 - partial agreement; 4 - full agreement. p values denote differences differences between each session mean.

**Table 5:** Willingness to adopt the system as a reference tool

**n**	**Item**	**Mean**	**SD**
15	I think this system could be used in other basic science subjects	3.77	0.43
16	I think this system could be used in clinical science subjects	3.32	0.75
17	I see an advantage in using this system as a tool in my daily study	3.26	0.71
18	I think this system would allow me to obtain results similar or better than my average results while investing less time studying	2.96	0.83
19	I wish this system would encompass the content in the way I am taught at school	3.51	0.62
20	I would like to create content to take advantage of it using this system	3.40	0.71
21	I would like to collaborate in real time with my colleagues to build useful content fast	2.94	0.63
22	I would like to be able to print the notebooks from the system	3.74	0.57
23	I would rather use this system instead of my regular notebooks provided all the required content is available	3.11	0.84
24	I would rather use this system instead of lecture materials provided all the required content is available	3.19	0.80
25	I would rather use this system instead of the recommended bibliography provided all the required content is available	3.11	0.89
26	I would recommend this system to my colleagues	3.66	0.52

SD - Standard deviation. Student agreement to each of the items was assessed using a 4-point likert scale: 1 - full disagreement; 2 - partial disagreement; 3 - partial agreement; 4 - full agreement.

## Conclusions

Overall the application brings a new set of tools that may be helpful to organize knowledge in meaningful ways as well as to manage study sessions, based on personal performance metrics. The system takes into consideration learning object design, instructional design guidelines and principles from cognitive learning theories. Specifically the system allows students to: (1) create personal and reusable learning materials in a collaborative on-line environment (2) self-assess their knowledge through spaced repetition of open ended questions (3) view detailed feedback on their performance and progress (4) easily use the feedback for deliberate practice and to tailor future learning experiences.

Assessment of student performance on content presented through this system and direct comparison of learning outcomes against other learning tools and methods are the aims of future work. The development of these features is an important step towards bringing information management tools to support study decisions and improving learning outcomes.

## Availability and requirements

**Project name:** ALERT STUDENT**Project home page:**http://www.alert-student.com**Operating systems:** Platform independent**Programming languages:** HTML, CSS, Javascript, Java, Oracle SQL**Other requirements:** Internet explorer 8+, Firefox, Google Chrome, Safari**License:** Not opensource**Any restrictions to use by non-academics:** No restrictions

## Abbreviations

ACT-R: Adaptive character of thought; CSCL: Computer supported collaborative learning; FMUP: Faculty of Medicine of the University of Porto; HTML: HyperText Markup Language; MeSH: Medical subjects headings; SVG: Standard vector graphics; S1: Study session 1; S2: Study session 2.

## Competing interests

The authors declare that they have no competing interests.

## Authors’ contributions

TTG conceived, designed and implemented the system, designed the study and wrote the manuscript. AS conceived and designed the system and wrote the manuscript. MJG oversaw and approved the overall operation for the system development. MS designed the study, performed the statistical analysis and revised the manuscript. MAF oversaw and approved the study design, and revised the manuscript. All authors read and approved the final manuscript.

## Pre-publication history

The pre-publication history for this paper can be accessed here:

http://www.biomedcentral.com/1472-6920/14/143/prepub
